# Atractylenolide-I restore intestinal barrier function by targeting the S100A9/AMPK/mTOR signaling pathway

**DOI:** 10.3389/fphar.2025.1530109

**Published:** 2025-03-24

**Authors:** Chen Chen, Bingjie Sun, Keming Chen, Han Bao, Yu Tao, Jinyong Zhou, Xiaomin Yuan, Linhai He, Zhihua Lu, Kaidi Chen, Yang Li, Chengli Yu, Yugen Chen, Yinan Zhang

**Affiliations:** ^1^ Nanjing University of Chinese Medicine, Nanjing, China; ^2^ Jiangsu Province Hospital of Chinese Medicine, The Affiliated Hospital of Nanjing University of Chinese Medicine, Nanjing, China; ^3^ Jiangsu Province Key Laboratory of Tumor Systems Biology and Chinese Medicine, The Affiliated Hospital of Nanjing University of Chinese Medicine, Nanjing, China; ^4^ Xuzhou City Hospital of Chinese Medicine, The Affiliated Hospital of Nanjing University of Chinese Medicine, Xuzhou, China

**Keywords:** atractylenolide-1, intestinal barrier, S100A9, tight junction, AMPK/mTOR

## Abstract

Impaired intestinal epithelial barrier function is closely associated with the pathogenesis of ulcerative colitis (UC). Atractylenolide-I (AT-I), a major sesquiterpene derived from the herb *Atractylodes macrocephala* Koidz., has been reported to alleviate DSS-induced colitis in mice. This study aims to investigated the protective effects of AT-1 on intestinal epithelial barrier function and elucidate it’s underlying mechanisms. *In vivo*, an acute colitis model was established in mice, and transcriptomic analysis to identify differentially expressed genes. *In vitro*, overexpression plasmids and recombinant protein were used to evaluate their effects on intestinal barrier function, and further analysis of its potential mechanisms.The study found that AT-1 ameliorate DSS-induced acute ulcerative colitis, exhibiting protective effects on the intestinal barrier. Transcriptomic analysis revealed that AT-1 significantly modulated the expression of S100A8 and S100A9. Further investigations indicated that S100A9, rather than S100A8, mediated the expression of tight junction proteins, meanwhile, AT-1 reduces neutrophil activation and subsequent release of S100A9. Mechanistically, recombinant human S100A9 protein was found to induce a decrease in intracellular Ca^2+^ concentration, while AT-1 regulated the expression of tight junction proteins *via* modulation of the AMPK/mTOR signaling pathway. AT-1 enhances the recovery of DSS-induced intestinal barrier dysfunction by regulating the recombinant human S100A9 protein-mediated AMPK/mTOR signaling pathway. This study provides new insights into the pathogenesis of ulcerative colitis and suggests potential therapeutic strategies for its treatment.

## Graphical Abstract

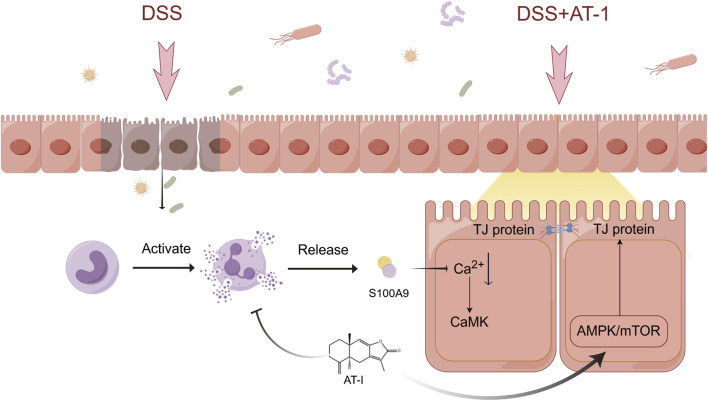

## 1 Introduction

Ulcerative Colitis (UC), a subtype of inflammatory bowel disease (IBD), remains elusive in terms of its etiology (Le Berre et al., 2023). Although the precise cause of UC is still undetermined, evidence suggests that it arises from multiple contributing factors, including epithelial barrier dysfunction, dietary Frontiers in Pharmacology habits, environmental influences, bacterial infections, autoimmune responses, and genetic predisposition (Du and Ha, 2020). Clinically, the treatment of UC primarily aims to relieve symptoms due to the short of definitive therapy. However, commonly used medications, such as 5-aminosalicylic acid, immunomodulators, anti-TNF-α monoclonal antibodies, and probiotics, have notable limitations in their effectiveness and applicability (Voelker, 2024), leading to extensive exploration of efficient, low-toxicity, and cost-effective treatment for UC.

The intestinal epithelial barrier serves as the first line of defense against harmful substances and pathogens present in the intestinal lumen, playing a vital role in maintaining the balance of gut microbiota and overall health (Mehandru and Colombel, 2021). Although the question of whether changes in the intestinal barrier precede the onset of inflammatory bowel disease (IBD) or result from inflammation remains controversial, recent research underscores the critical importance of intestinal barrier function. For example, a key clinical study published in 2020 demonstrated that intestinal barrier damage may occur years prior to the clinical diagnosis of IBD, and intestinal permeability is recognized as a significant predictive indicator of relapse risk in IBD patients (Turpin et al., 2020). The structural integrity of the intestinal barrier relies on intact epithelial cells and the tight junctions between these cells, which consist of various closely packed protein complexes, including ZO-1, occludin, and members of the claudin family (Otani and Furuse, 2020). Importantly, the intestinal barrier is dynamic, constantly undergoing a balance of wear and repair. When this balance is disrupted over a prolonged period, it may precipitate the onset and progression of disease. Consequently, the development of targeted drugs aimed at enhancing intestinal barrier function has emerged as a promising area for treating UC (Odenwald and Turner, 2017).

Our group is dedicated to studying the pharmacological mechanisms of naturally-abundant constituents that existed in the traditional Chinese medicinal (TCM) herbs in the treatment of intestine disorders (Lv et al., 2021a; Lv et al., 2021b; Lv et al., 2021c). *Atractylodes macrocephala* Koidz. is a member of the Asteraceae family and the Atractylodes DC., commonly used in TCM for treating gastrointestinal disorders. This medicinal herb is known for its various therapeutic effects, modern pharmacological studies have demonstrated that *A. macrocephala* possesses anti-inflammatory, anti-tumor, immune-modulating, gastrointestinal function improvement and others (Zhu et al., 2018; Luo et al., 2024). Recent studies have demonstrated that atractylenolide-1 (AT-1), one of the main constituents in the plant extract, alleviates DSS-induced colitis in mice through the SPHK1/PI3K/AKT signaling pathway (Qu et al., 2022). However, the pharmacological effects of AT-1 were not investigated *in vitro* in this study. Furthermore, the dosages (50 mg/kg) used in the study also exceed the concentration ranges of the herbal material in clinical use. Given the critical role of intestinal epithelial barrier damage in the pathogenesis of ulcerative colitis (UC), study herein investigated the protective effects of AT-1 on the intestinal epithelial barrier and elucidate its potential molecular mechanisms. These data support the stabilization of intestinal barrier as an innovative strategy to fulfill the urgent need for intestine disorders.

## 2 Materials and methods

### 2.1 Chemicals and reagents

Atractylenolide-I (AT-1, C15H18O2, MW:230.32, ≥98% purity) was purchased from Chengdu Push Biotech (Chengdu, China); sodium butyrate (NaB, C4H7NaO2, MW:110.09, ≥98% purity) was purchased from Macklin Biochemical Technology (Shanghai, China); dextran sulfate sodium (DSS) was purchased from MP Biomedicals (Solon, OH, USA); myeloperoxidase (MPO) Kit was purchased from Jiangsu Meimian industrial Co., Ltd (Nanjing, China); fluorescein isothiocyanate-labeled dextran (FD4, average mol wt 3,000–5,000) was purchased from Sigma Aldrich (St. Louis, MO, USA); recombinant human S100A9 protein, dorsomorphin and BAPTA-AM were purchased from Med Chem Express (Shanghai, China); ZO-1, occludin, claudin-1, MLCK, β-actin, HA, mTOR, AMPK-α, ERK1/2, phospho-ERK1/2 (Thr202/Tyr204), p38 MAPK, phospho-p38 MAPK (Thr180/Tyr182), Flag primary antibodies, HRP-conjugated Affinipure Goat Anti-Mouse IgG(H+L), HRP-conjugated Affinipure Goat Anti-Rabbit IgG(H+L) were purchased from Proteintech (Wuhan, China); SAPK/JNK, phospho-SAPK/JNK (Thr183/Tyr185), phospho-AMPKα (Thr172), myosin light chain 2, phospho-myosin light chain 2 (Thr18/Ser19) primary antibodies were purchased from Cell Signaling Technology (Danvers, MA, USA); rhodamine (TRITC) Affinipure goat anti-mouse IgG(H+L), FITC-AffiniPure rabbit anti-rabbit IgG (H+L) were purchased from Yeasen Biotechnology (Shanghai, China); DMEM, RPMI 1640 medium, pen/strep, fetal bovine serum (FBS), opti-MEM medium were purchased from Gibco BRL (Gaithersburg, MD, USA); total protein extraction kit were purchased from KeyGenbio (Nanjing, China); fastPure complex tissue/cell total RNA isolation kit, hiScript™ QRTSuperMix and AceQ™qPCR SYBR green master mix were purchased from Vazyme Biotech (Nanjing, China); lipofectamine™ 3000 transfection reagent were purchased from Thermo Fisher Scientific (Waltham, MA, USA); quickBlock™ blocking buffer for immunol staining was purchased from Beyotime (Shanghai, China); PBS, rapid sealing solution (Protein free) were purchased from Servicebio (Wuhan, China); other chemical products used were of the analytical grade available.

### 2.2 Animals, establishment and treatment of UC

The female C57BL/6 mice (weight, 20–22 g; age, 6–8 weeks) were purchased from the spfbiotech (Beijing) Biotechnology Co., Ltd. They were housed under a pathogen-free condition with the temperature of 23° ± 2°C and a relative humidity of 50%–55%, *ad libitum* to feed and water. Animal welfare and experimental procedures were strictly carried out in accordance with the Guide for the Care and Use of Laboratory Animals (National institutes of Health, USA). The protocol was approved by the Animal Ethics Committee of Nanjing University of Chinese Medicine, the ethics numbers are 202311A013 (application data is 21 November 2023, and the total number of mice is sixty).

DSS-induced colitis was performed as previously described. The female C57BL/6 mice were fed with 2.5% DSS in the drinking water for 7 days followed by 3 days of recovery. The mice were randomly divided into the following six groups (*n* = 7 in each group): blank group, DSS group, AT-I (1, 3, 10 mg/kg, i.p) group, and NaB (200 mg/kg, i,g) group. *In vivo* experiments, AT-I and positive control NaB were suspended in contain 1% DMSO and 1% tween-80 normal saline. The body weight, diarrhea and rectal bleeding were measured every day, and the disease activity index (DAI) scores were calculated using the well-established system. On day 10, mice were sacrificed, and their colons were gathered and photographed.

### 2.3 Cell culture

NCM460 cells and Caco-2 cells were purchased from the Cellverse Bioscience Technology Co., Ltd. (Shanghai, China). NCM460 cells were maintained in RPMI 1640 medium supplemented with 10% fetal bovine serum, 100 U/mL pen/strep. Caco-2 cells were maintained in DMEM medium supplemented with 20% fetal bovine serum, 100 U/mL pen/strep. Cells were cultured in a humid environment at 37°C and 5% CO2.

*In vitro* experiments, AT-I were dissolved in DMSO (the final concentration ≤0.1%) and diluted with culture medium to obtain the desired concentrations, the 0.1% DMSO was used as the control.

### 2.4 Permeability measurement

On the last day of DSS-induced colitis, 200 μL fluorescein isothiocyanate-dextran 4000 (FD4, 20 mg/mL) was orally administered to mice. After 6 h, blood was collected from the orbital venous plexus of mice and serum FD4 levels were measured using a fluorescence enzyme-linked immunosorbent assay (Synergy HT; BioTek, USA) at excitation and emission wavelengths of 485 nm and 530nm, respectively.

### 2.5 Histological analysis

The colons isolated from mice were fixed in Carnoy’s fluid for 24 h. Then, they were dehydrated, embedded in paraffin, and sliced into 4 μm slices, and stained with hematoxylin and eosin (H&E) for histological evaluation. The results were imaged using electron optical microscopy to capture the tissue morphology.

### 2.6 Transmission electron microscopy (TEM) examination

Colon tissue samples (approximately 1 mm^3^) were fixed in electron microscopy fixative overnight at 4°C, rinsed in PBS, and postfixed in 1% osmic acid for 2 h at room temperature. Dehydrated through a graded acetone series and embedded in epoxy resin, ultrathin sections (80–100 nm) were cut and double-stained with 2% uranium acetate and lead citrate. The ultrastructure of colon epithelial tight junctions was then visualized using transmission electron microscopy.

### 2.7 Alcian blue staining

The tissue samples were prepared into 5 micron thick paraffin sections, and the paraffin was removed according to standard procedures. Then, the sections were immersed in the alcian blue staining solution for 15 min, followed by thorough washing with distilled water to stain the nucleus. After completing the staining process, the sections were subjected to a progressive dehydration treatment and sealed with neutral resin. Finally, images were captured using a vertical brightfield microscope.

### 2.8 Immunohistochemical staining

Immunohistochemical staining of colon tissue sections, derived from paraffin-embedded samples, was conducted following standard protocols. The tissue sections were first incubated with primary antibodies specific to ZO-1, occludin, and claudin-1. This was followed by a secondary antibody incubation, strictly adhering to the guidelines provided with the kit. The presence of the peroxidase conjugate was then revealed using a diaminobenzidine solution, which produces a visible color reaction at the antigen sites. The sections were then counterstained with hematoxylin and mounted on a coverslip. Finally, the images were gained using a light microscope.

### 2.9 Immunofluorescence staining

To perform immunofluorescence staining on colon tissues, we prepared 5 μm-thick sections and treated them with an immunohistochemistry blocking solution to prevent non-specific binding. These sections were then incubated with anti-MUC2 antibodies at 4°C overnight. After three rounds of washing with PBS, the slides were further incubated with secondary antibodies conjugated to a fluorescent marker at room temperature for a duration of 2 h. Subsequently, the sections were stained with DAPI at a concentration of 0.1 μg/mL for 10 min to visualize the nuclei. As a control, sections were incubated with secondary antibodies alone to serve as a negative control.

For cellular staining, cells grown on coverslips were fixed using a 4% paraformaldehyde solution at room temperature for 30 min and subsequently washed with PBS. To block any non-specific binding sites, the cells were treated with an immunohistochemistry blocking solution for 1 h at 37°C. They were then incubated with the primary antibody at 4°C overnight. Following multiple washes with PBS, the cells were incubated with secondary antibodies—either goat anti-rabbit or anti-mouse IgG conjugated to FITC or rhodamine—for 1 h. The cells were then stained with DAPI at a concentration of 0.1 μg/mL for 10 min to highlight the nuclei. Finally, the stained cells were captured using a fluorescence microscope.

### 2.10 Western blot analysis of protein expression

The total proteins in the colon tissues of mice with DSS-induced colitis or cells were extracted using total protein extraction kit. Then, the proteins were separated using 8%–15% SDS-PAGE, and transferred to 0.22 μm PVDF membranes. The membranes were blocked with Quick sealing solution (protein free) for 10 min, and incubated overnight at 4°C with specific primary antibodies, and then incubated with HRP-conjugated secondary antibody for 1 h at 37°C. Detection was performed by the Tanon 5200 Multi Fully Automatic Chemiluminescence/Fluorescence Image Analysis System.

Colonic tissues from mice with DSS-induced colitis and cellular lysates were processed for total protein extraction using a commercial kit. Proteins were resolved by 8%–15% SDS-PAGE and electroblotted onto 0.22 μm PVDF membranes. After a 10-min blocking with a protein-free reagent, membranes were probed with specific primary antibodies at 4°C overnight, followed by incubation with HRP-conjugated secondary antibodies for 1 h at 37°C. Chemiluminescent signals were detected using the Tanon 5200 Multi Fully Automatic Chemiluminescence/Fluorescence Image Analysis System.

### 2.11 Quantitative real-time PCR

Total RNA from colon tissues or cell pellets was extracted using FastPure Complex Tissue/Cell Total RNA Isolation Kit according to the manufacturer’s instructions, and its purity and concentration were determined by measuring and comparing the absorbance at 260 nm and 280 nm. Then, 1 µg of total RNA was reversely transcribed to cDNA, and using AceQ™qPCR SYBR Green Master Mix in ABI 7500 real-time fluorescence quantitative for RT-PCR. The primer sequences used were listed in Table 1. The expression of each gene was normalized to *Gapdh*, and calculated using 2^−ΔΔCT^ method.

**TABLE 1:** The primers used in qPCR assay.

Primers		Sequence (5′- 3′)
Gapdh (mouse)	Forward	GGT​GAA​GGT​CGG​TGT​GAA​CG
	Reverse	CTC​GCT​CCT​GGA​AGA​TGG​TG
Il1b (mouse)	Forward	GCT​GTG​GAG​AAG​CTG​TGG​CA
	Reverse	TGG​GAA​CGT​CAC​ACA​CCA​GC
Tnf (mouse)	Forward	CCC​TCA​GCG​AGG​ACA​GCA​AG
	Reverse	ACA​GAA​CCT​GCC​TGG​TTG​GC
Il10 (mouse)	Forward	AAG​GCA​GTG​GAG​CAG​GTG​AA
	Reverse	CCA​GCA​GAC​TCA​ATA​CAC​AC

### 2.12 Transepithelial electrical resistance (TEER) measurement

Caco-2 cells were cultured in Transwell inserts with 0.4 μm pores (Corning, Tewksbury, MA) for 21 days to achieve polarized monolayers. Prior to challenge, cells were exposed to AT-1 (2.5, 5, 10 µM) or NaB (200 µM) for 1 h. Subsequently, they were treated apically with LPS (1 μg/mL) or recombinant human S100A9 protein (5 ng/mL) for 24 h. Post-treatment, the monolayers were rinsed with Hank’s balanced salt solution (HBSS), and the apical medium was substituted with FD4 (100 μg/mL) in HBSS. Following a 1-h incubation at 37°C, the fluorescence intensity in the basal chamber was quantified using a plate reader set at 485 nm for excitation and 530 nm for emission. Fluorescein concentrations were ascertained relative to a standard curve.

Fluorescence transmittance (%) = FD4 concentration in the lower chamber/FD4 concentration added to the upper chamber.

The electrical resistance of intestinal monolayer cells (Caco-2 and HT-29 cells) was measured using a Millicell ERS-2 Voltohmmeter (Millipore) and calculated as follows: (resistance of treated cells (Ω) - resistance of blank well (Ω)) × effective membrane area (0.33 cm^2^) normalized to the control (untreated cells). During the TEER measurements, the apical and basolateral surfaces of the cell monolayers were immersed in culture medium. The electrical resistance was assessed until three consecutive readings produced consistent values, ensuring the stability of the monolayer.

### 2.13 MPO activity

The colon tissues of mice were homogenized with PBS, centrifuged at 3000 rpm for 15 min, and the supernatants were collected. The activity of MPO was measured using Myeloperoxidase (MPO) Kit according to the manufacturer’s instructions.

### 2.14 The transfection in NCM460 cells

Plasmid constructs were generated by inserting PCR-amplified S100A8, and S100A9 into pcDNA vectors (Corues Biotechnology Co., Ltd., Nanjing), with subsequent verification by DNA sequencing. NCM460 cells were cultured in six-well plates at a seeding density of 1 × 10^5^cells/mL. Transfection was executed in accordance with the manufacturer’s protocol, utilizing 3 μg of either plasmid DNA or an empty vector to transfect cells that had reached 70%–80% confluence. The transfection complex was formed with 3 μL of Lipofectamine 3000 and 6 μL of p3000 reagent and applied to cells in Opti-MEM medium. The success of transfection was evaluated 36 h later under a fluorescence microscope before proceeding with further experimental procedures.

### 2.15 Flow cytometry

After incubating S100A9 for 24 h with NCM460 cells, the cells were digested with trypsin and collected. The cells were incubated in 1 μM Fluo-4/AM-containing PBS without calcium in the dark for 40 min. The cells were washed with PBS twice, and then placed in a flow cytometer for analysis by flow cytometry.

### 2.16 Statistical analysis

All statistical analyses were performed using GraphPad Prism 8 software. All data are expressed as the mean ± S.D. values. One-way ANOVA followed by LSD test was used to compare the mean differences between multiple groups. A difference was considered significant if the *p*-value was <0.05.

## 3 Result

### 3.1 AT-1 alleviates DSS-induced colitis in mice

**FIGURE 1:**
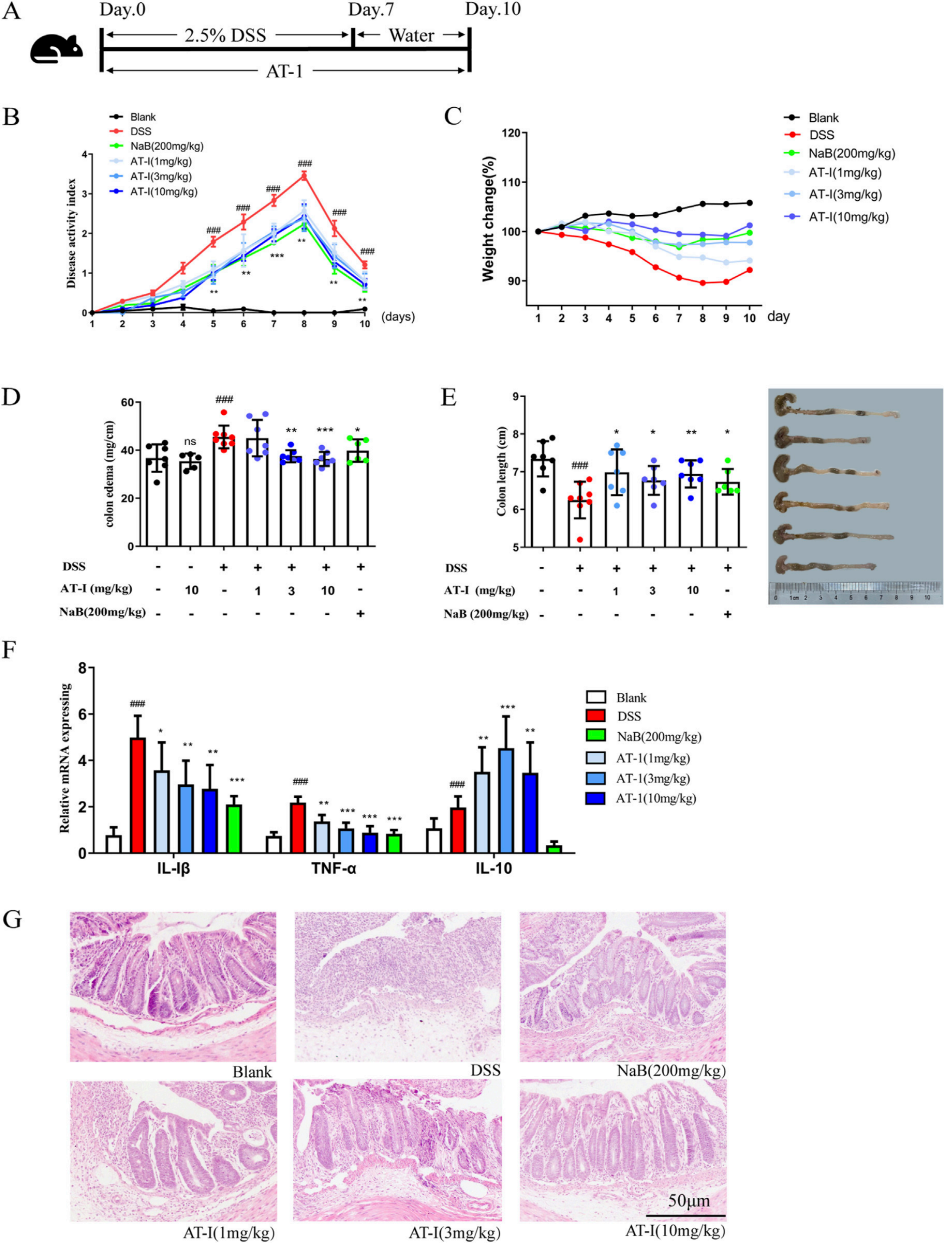
AT-1 attenuated DSS-induced experimental colitis. The mice were fed with 2.5% dextran sulfate sodium (DSS) for 7 days, and then given normal drinking water for 3 days. AT-Ⅰ(1, 3, 10 mg/kg) and NaB (200 mg/kg) were administered daily for 10 consecutive days. At the end of the experiment, the mice were sacrificed, the colons were collected. **(A)** Experimental process, **(B)** the disease activity index (DAI), **(C)** the body weight change, **(D)** the Intestinal/weight index, **(E)** the colon length, **(F)** The mRNA levels of *Tnf, Il1b* and *Il10* genes in colon tissues were determined by real-time qPCR assay, **(G)** the histopathological changes of colons were detected by using H&E staining (scale bar: 50 μm), *n* = 6–7. ^##^ *p* < 0.01 vs. Blank group; ^***^ *p* < 0.001 and ^***^ *p* < 0.001 vs. DSS group.

As shown in Figure 1A, female C57BL/6 mice were subjected to treatment with 2.5% DSS for 7 days, followed by normal drinking water for an additional 3 days to establish an acute colitis model. AT-1 (1, 3, and 10 mg/kg) and sodium butyrate (NaB, 200 mg/kg) were administered orally on a daily basis for 10 days. The Disease Activity Index (DAI) scores were recorded according to established protocols (Lv et al., 2021a), and the results are presented in Figure 1B. Compared to the DSS group, AT-1 significantly reduced DAI scores in a dose-dependent manner. Furthermore, after transitioning to normal drinking water, the AT-1 (10 mg/kg) group exhibited a more rapid recovery in body weight compared to the NaB (200 mg/kg) group (Figure 1C). Inflammation leads to colon shortening and edema, and the colon length-to-weight ratio serves as a visual representation of the overall inflammatory state in the mouse colon (Nakamura et al., 2021). Treatment with AT-1 (3 and 10 mg/kg) and NaB (200 mg/kg) resulted in lower inflammation levels, while AT-1 alone did not incite colon inflammation (Figure 1D). Additionally, AT-1 mitigated colon shortening induced by DSS treatment (Figure 1E). Moreover, the AT-1 group exhibited a marked reduction in the expression of pro-inflammatory factors, IL-1β and TNF-α mRNA, in the colons of UC mice, while significantly increasing the expression of the anti-inflammatory factor IL-10 mRNA (Figure 1F). Histological analysis revealed that mice treated with AT-1 (10 mg/kg) experienced significantly reduced mucosal damage, diminished inflammatory cell infiltration, and less crypt loss (Figure 1G). Importantly, the AT-1 (10 mg/kg) treatment significantly increased the number of intact colonic crypts, comparable to the effects of NaB, indicating that AT-1 has a protective role in maintaining colonic epithelial integrity.

### 3.2 AT-1 alleviates intestinal barrier injury in DSS-induced colitis mice

**FIGURE 2:**
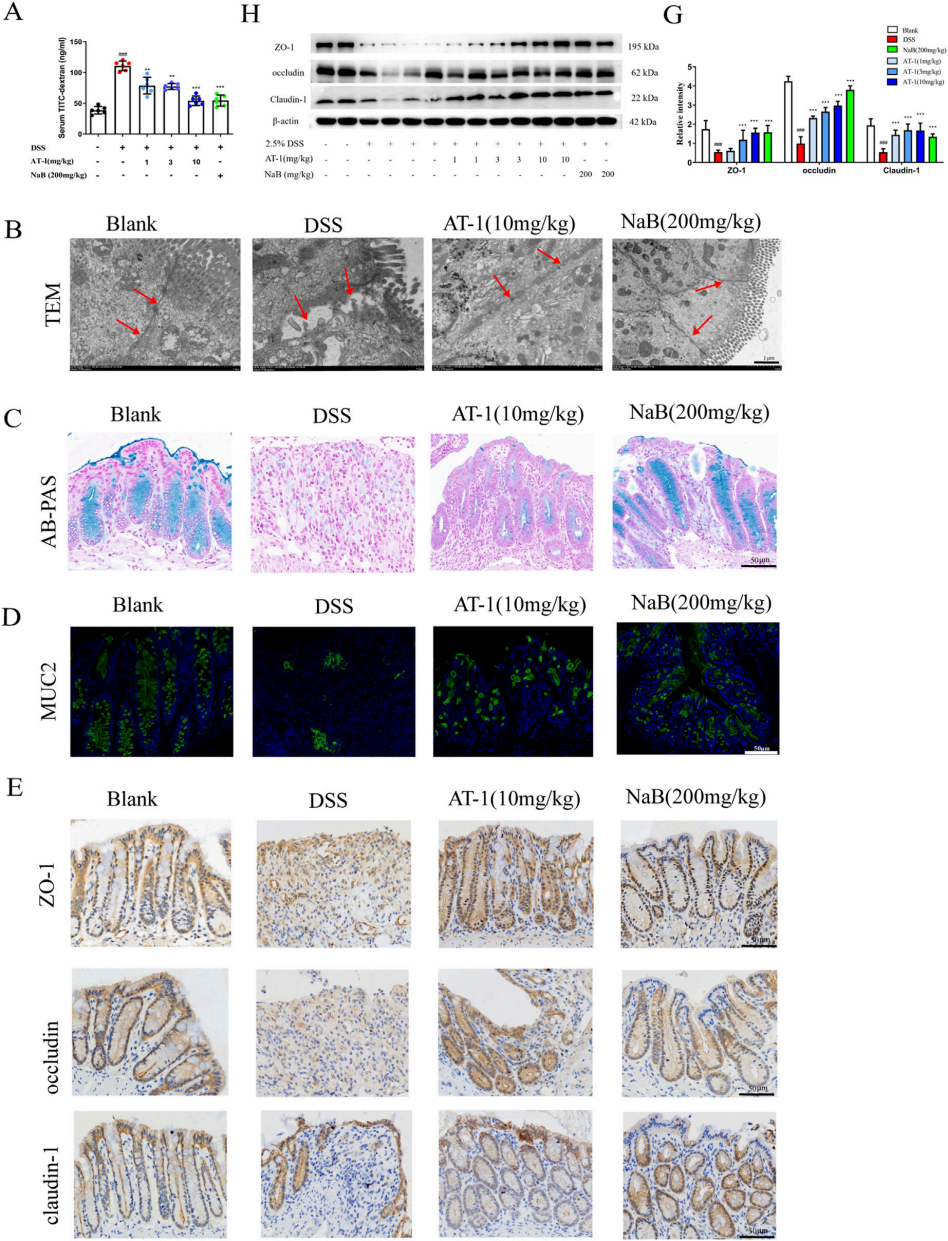
AT-1 ameliorate DSS-induced the intestinal barrier in mice. **(A)** Quantification of paracellular FD4 passage across intestinal epithelial barrier in mice (*n* = 6–7), **(B)** Ultrastructure of tight junction in colon tissue as observed by transmission electron microscopy (arrows indicate epithelial barrier integrity, scale bar: 1 µM), **(C)** Alcian blue staining was used to observe intestinal mucus levels in colon(scale bar: 50 µM), **(D)** Immunofluorescence staining was used to detect the expression of MUC2 in colon(scale bar: 50 µM), **(E)** Immunohistochemistry staining was used to detect the expression of ZO-1, occludin, claudin-1 in colon (scale bar: 50 µM), **(H–G)** Effects of AT-1 on protein expression of ZO-1, occludin, and CLDN1 in colon tissues of mice were determined by Western blot(*n* = 6). ^##^ *p* < 0.001 vs. Blank group; ^*^ *p* < 0.05, ^**^ *p* < 0.01 and ^***^ *p* < 0.001 vs*.* DSS group.

To evaluate the impact of AT-1 on epithelial barrier function in the DSS-induced ulcerative colitis model, we assessed intestinal permeability in mice using fluorescein isothiocyanate-dextran (FITC-dextran, FD4). As shown in Figure 2A, serum FD4 content in the DSS group was significantly higher than that in the blank group. In contrast, both the AT-1 and NaB groups exhibited reduced serum FD4 levels, indicating that AT-1 effectively decreased intestinal permeability. Transmission electron microscopy observations (Figure 2B) revealed that colon samples from DSS group mice displayed significant intercellular spaces and loss of tight junctions. Conversely, treatment with AT-1 and NaB significantly improved these morphological alterations.

Given that AT-1 was administered *via* intraperitoneal injection and had no direct contact with the intestinal microbiota, we first examined its effect on the chemical barrier. Colonic mucus secretion was evaluated using the Alcian Blue staining method. The results presented in Figure 2C indicate that mice in the DSS group exhibited nearly complete loss of colonic mucus, alongside crypt structure damage. Both AT-1 and NaB alleviated the thinning of colonic mucus caused by DSS. Further tissue immunofluorescence staining for MUC2 protein (Figure 2D) demonstrated that, compared to the DSS group, both the AT-1 and NaB treatment groups upregulated MUC2 protein expression. However, it is noteworthy that while the crypt structure in the AT-1 treatment group remained relatively intact, the quantity of mucus and the fluorescence intensity of the main mucus layer protein MUC2 were lower than in the blank and NaB groups. The method of administration and the results regarding MUC2 protein indicate that the mechanism of AT-1 may be associated with the maintenance of epithelial barrier integrity.

To verify our hypothesis, immunohistochemical analysis (Figure 2E) of tight junction proteins ZO-1, occludin, and Claudin-1 were studied to show significantly reduction in the colon tissue of DSS group mice, coinciding with crypt structure loss. Following interventions with AT-1 (10 mg/kg) and NaB (200 mg/kg), the expression levels of these tight junction proteins significantly increased in colon. Western blot analysis (Figures 2F,G) corroborated the immunohistochemical findings and the efficacy of AT-1 on the physical barrier.

These results indicate that AT-1 shows better efficacy on the physical barrier, significantly improving the loss of tight junctions induced by DSS, thereby maintaining the integrity of the intestinal epithelial barrier function.

### 3.3 mRNA-seq analysis of the colon tissue of mice with colitis treated with AT-I

**FIGURE 3:**
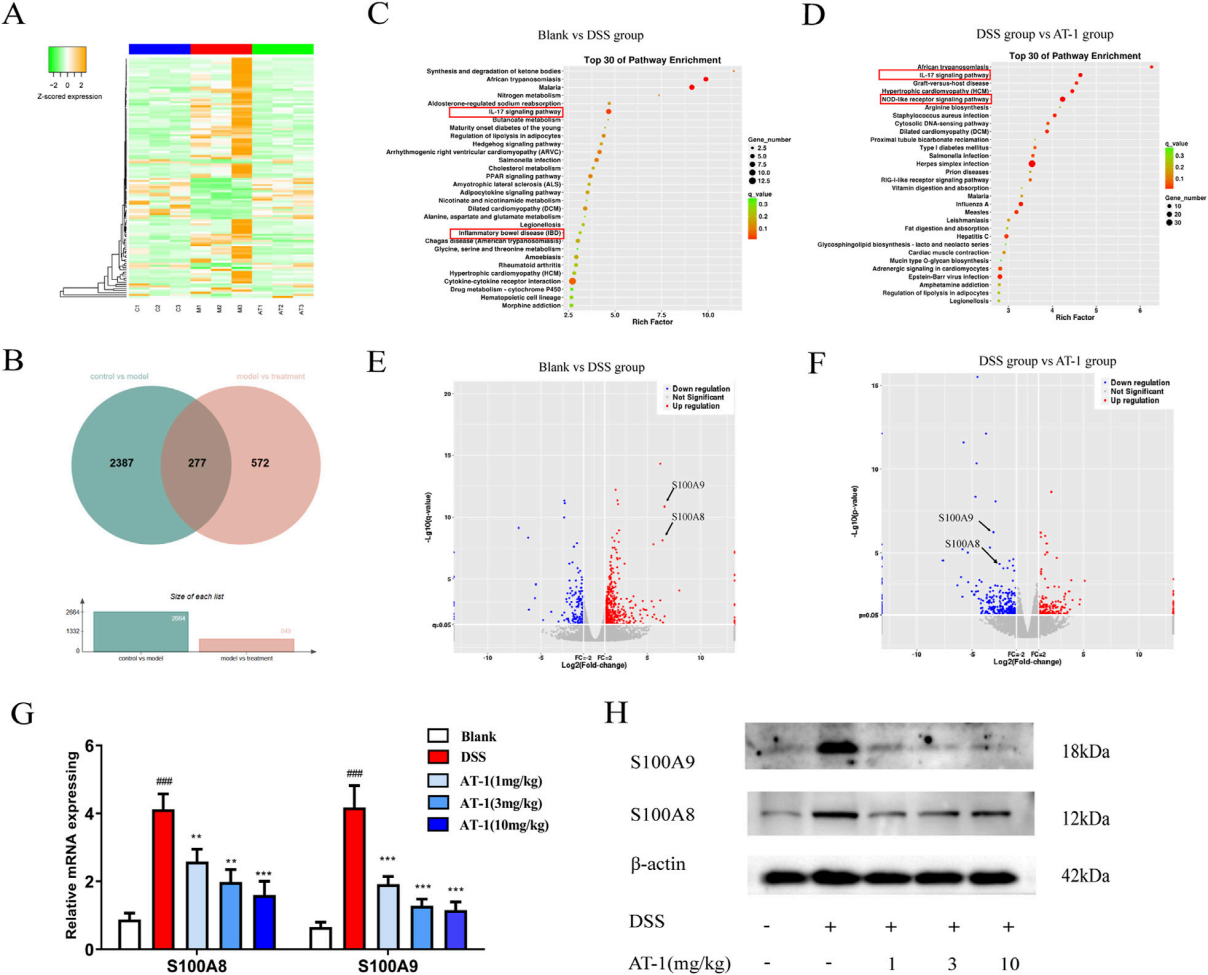
Transcriptomic analysis of AT-1 treated DSS-induced colitis in mice. **(A)** Differential gene heatmap analysis of each group. **(B)** Differential gene Venn diagram of blank group, DSS group, and AT-1 group. **(C,D)** Differential gene volcano map of each group. **(C)** blank group vs. DSS group, **(D)** DSS group vs. AT-1 group. **(E,F)** Differential gene volcano map of each group, **(E)** blank group vs. DSS group, **(F)** DSS group vs. AT-1 group. G-H Expression levels of S100A8/S100A9 mRNA **(G)** and protein **(H)** in mice colon tissue. ^###^ *p* < 0.001 vs. Blank group; ^**^ *p* < 0.01 and ^***^ *p* < 0.001 vs*.* DSS group.

To explore the molecular mechanisms, we performed mRNA-seq analysis on mouse colonic mucosal samples (For all differentially expressed genes, please refer to Supplementary Table S1). The sequencing results revealed that, compared to the blank group, 293 genes exhibited significantly increased expression levels in the DSS group, which were downregulated following AT-1 treatment. Conversely, 179 genes showed significantly lower expression levels in the DSS group, which were significantly upregulated after AT-1 administration (Figures 3A,B). The pathogenic mechanisms of ulcerative colitis (UC) are unclear, but it is currently believed to be associated with genetic factors, abnormal activation of immune cells, dysbiosis of gut microbiota, and changes in gut permeability (Le Berre et al., 2023). GO analysis indicated that 492 genes regulated by AT-1 treatment were closely associated with immune processes, including the IL-17 signaling pathway, Nod-like receptor (NLR) signaling pathway, and arginine biosynthesis (Figures 3C,D). Additionally, in the comparison between the model group and the treatment group, the IL-17 signaling pathway ranked higher, with more genes being enriched. It is important to note that the higher rich factor for African trypanosomiasis is not related to the pathogenesis of UC (Streit and Matsumoto, 2016; Pays et al., 2023).

Further differential gene analysis in IL-17 signaling pathway highlighted (Figures 3E,F) that AT-1 significantly modulated the transcription levels of S100A8/S100A9 in the colons of UC mice. To validate the mRNA-seq results, we conducted additional experiments on mouse colon tissue. QT-PCR and Western blot analysis (Figures 3G,H) revealed that AT-1 significantly reduced S100A8/S100A9 expression at both the mRNA and protein levels. This finding indicates that AT-1 may mitigate intestinal barrier dysfunction by downregulating S100A8/S100A9 expression.

### 3.4 AT-I ameliorate the expression of tight junction proteins induced by S100A9

**FIGURE 4:**
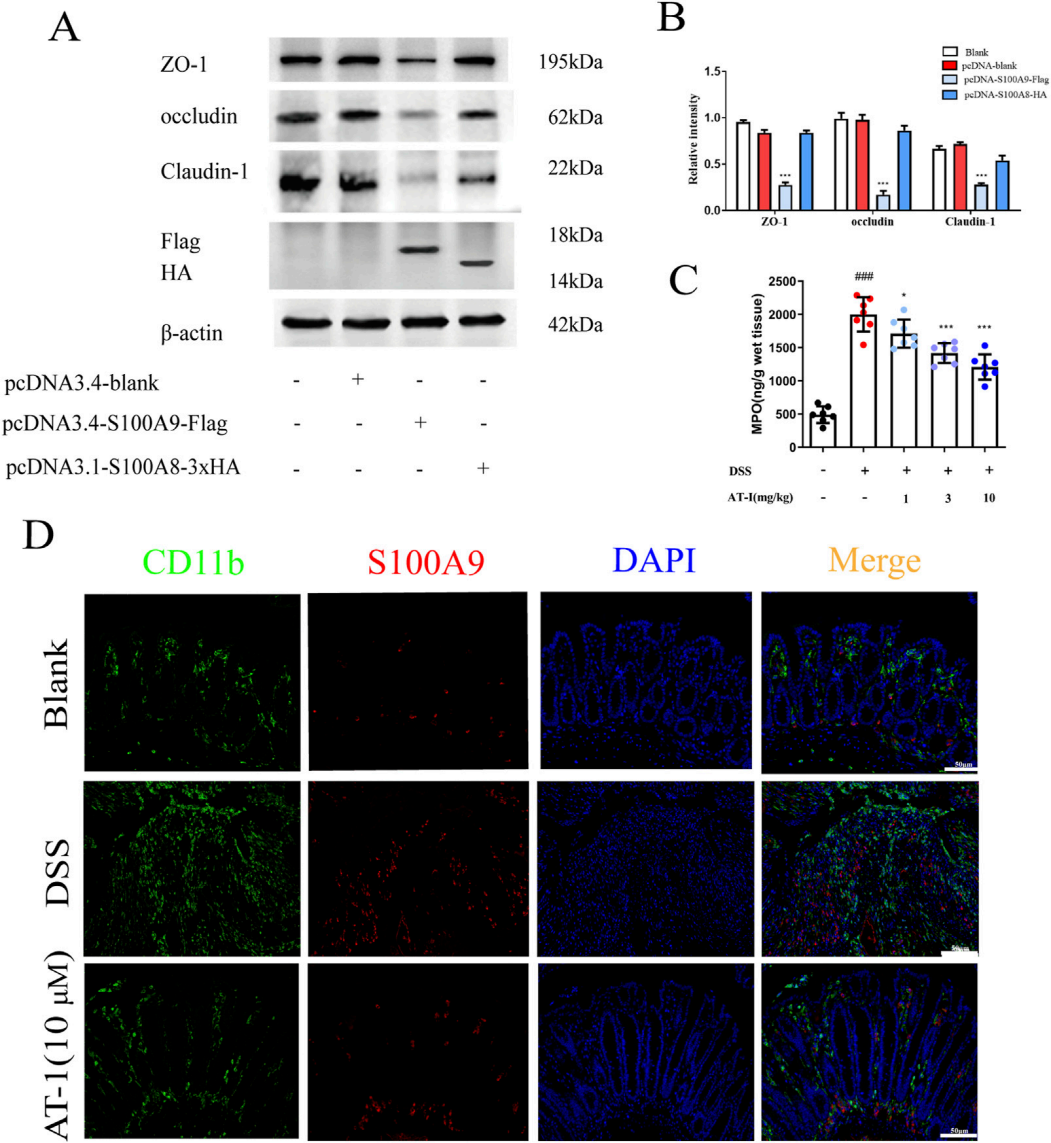
AT-1 reduces neutrophil activation and S100A9 release in DSS-induced colitis. **(A,B)** NCM460 cells, transfected with pcDNA-Flag, pcDNA-S100A8-HA, or pcDNA-S100A9-Flag, were analyzed by Western blot after 36 h **(C)** the MPO activity in colons were measured. **(D)** CD11b^+^ and S100A9 in colonic tissue were determined by using immunofluorescence assay (scale bar: 50 μm). ^###^ *p* < 0.001 vs. Blank group; ^*^ *p* < 0.05 and ^***^ *p* < 0.001 vs*.* DSS group or pcDNA group.

Previous studies have suggested that S100A8 and S100A9 form a heterodimer *in vivo*, participating in various biological activities both intracellularly and extracellularly (Song and Struhl, 2021; von Wulffen et al., 2023). To assess the impact of S100A8/S100A9 on intestinal barrier function, we overexpressed S100A8 and S100A9 separately in the colon epithelial cell line NCM460 to evaluate their effects on tight junction protein expression. Interestingly, when NCM460 cells were transfected with either the S100A8 or S100A9 plasmids, significant alterations were observed, our results (Figures 4A,B) indicated that S100A9, rather than S100A8, disrupted the expression of tight junction proteins in intestinal epithelial cells. S100A9 is predominantly released by neutrophils (Pruenster et al., 2023), and MPO serves as an indicator for assessing the quantity and activity of neutrophils. ELISA analysis demonstrated that, compared to the DSS group, the concentration of MPO in the colon tissue of mice treated with AT-1 was significantly reduced (Figure 4C). Furthermore, immunofluorescence staining (Figure 4D) demonstrated that AT-1 decreased the aggregation of neutrophils marked by CD11b^+^ in the intestine and S100A9 showed less co-localization with CD11b^+^ neutrophils.

**FIGURE 5:**
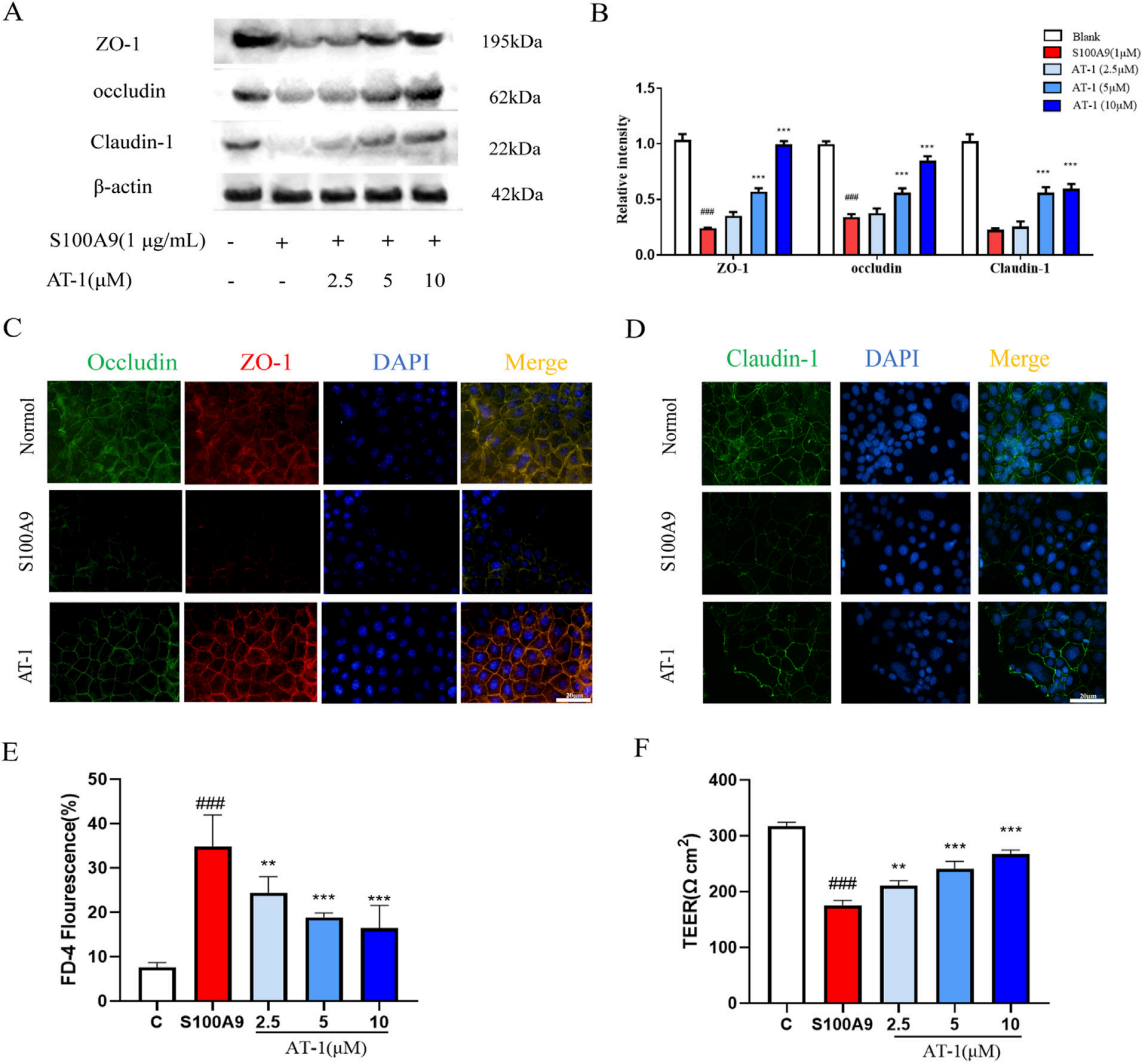
AT-1 protected expression of TJ protein and barrier function of colonic epithelial cells caused by S100A9. **(A,B)** NCM460 cells were pretreated with different concentrations of AT-1 for 1 h and then stimulated with S100A9 (1 μg/mL) for 24 h **(C,D)** Paracellular permeability and TEER of Caco-2 cells stimulated with S100A9 (1 μg/mL) for 24 h with AT-1 (2.5, 5, 10 µM) treatment. **(E,F)** Immunofluorescence staining was used to examine the distributions of TJ proteins after exposure to S100A9 (1 μg/mL) in the presence or absence of AT-1 in Caco-2 cells (scale bar: 20 µM). ^###^ *p* < 0.001 vs. Blank group; ^**^ *p* < 0.01 and ^***^ *p* < 0.001 vs*.* S100A9 group.

To further investigate whether AT-1 could ameliorate the effects of S100A9 on tight junction proteins, we treated cells with recombinant human S100A9 protein *in vitro* and assessed the efficacy of AT-1.As shown in Figures 5A,B, AT-1 treatment group dose-dependently reversed the reduction in the expression of ZO-1, occludin, and claudin-1 in NCM460 cells induced by the recombinant human S100A9 protein (1 μg/mL), which was also confirmed by the immunofluorescence of the recombinant human S100A9 protein on tight junction proteins (Figures 5C,D). Moreover, we utilized a monolayer cell structure differentiated from Caco-2 cells to examine the effect of AT-1 on barrier function. The results (Figures 5E,F) demonstrated that the recombinant human S100A9 protein (1 μg/mL) decreased the transepithelial electrical resistance of the monolayer cells and increased intercellular permeability, while AT-1 was able to reverse these changes.

### 3.5 AT-1 modulates AMPK/mTOR signaling pathway mediated by recombinant human S100A9 protein

**FIGURE 6:**
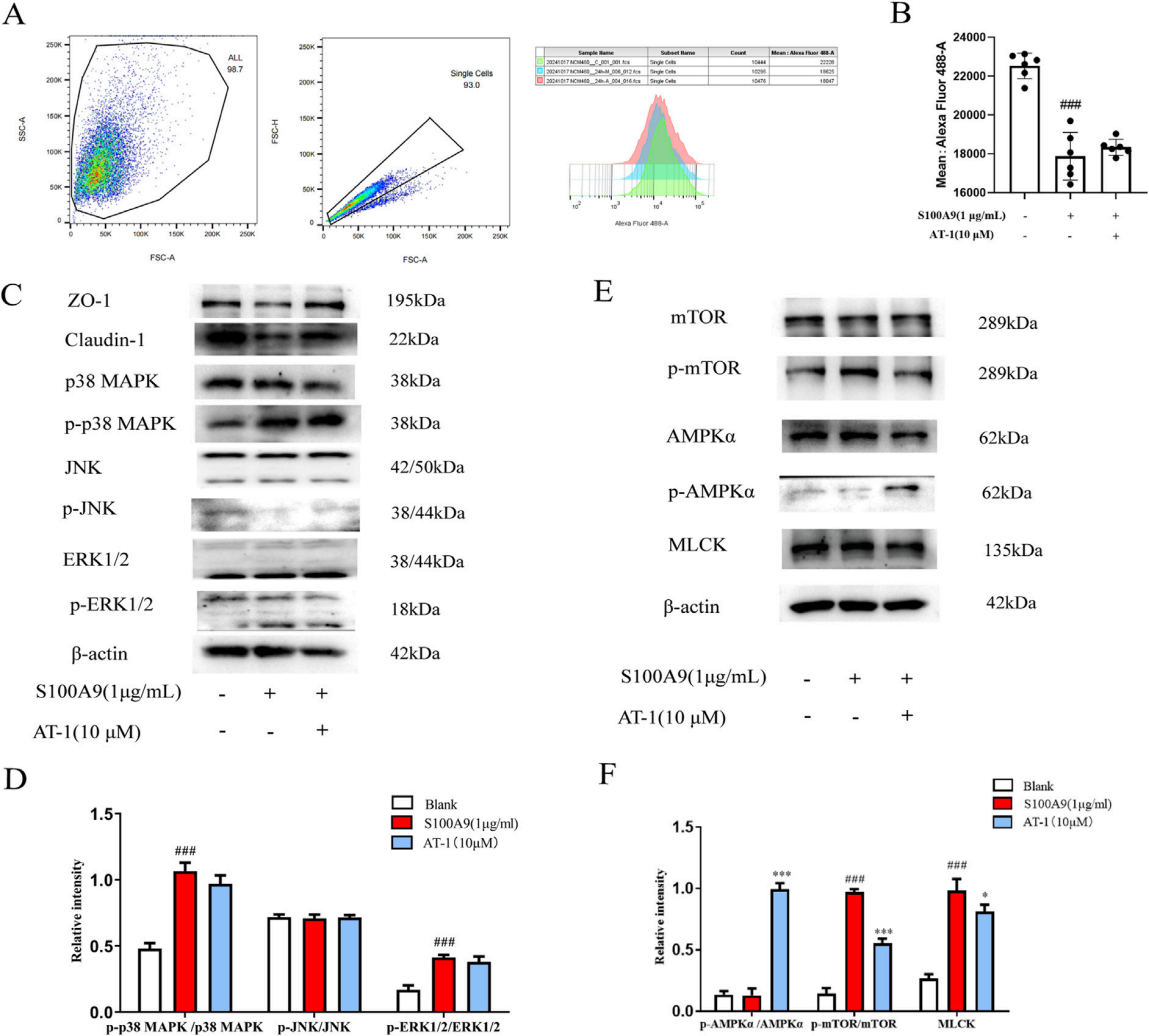
S100A9 regulated the expression of TJ proteins in colonic epithelial cells through AMPK/mTOR pathway. **(A,B)** NCM460 cells were pretreated with AT-1 (10 μM) for 1 h, followed with S100A9 (1 μg/mL) for 24 h, then detected by flow cytometry assay. **(C–F)** NCM460 cells were pretreated with AT-1 (10 μM) for 1 h, followed with S100A9 (1 μg/mL) for 24 h, the level of ZO-1, claudin-1, p38MAPK, p-p38MAPK, JNK,p-JNK, ERK1/2, p-ERK1/2, AMPKα, p-AMPKα, mTOR, p-mTOR, and MLCK, was evaluated by Western blot. ^###^ *p* < 0.001 vs. Blank group; ^*^ *p* < 0.05 and ^***^ *p* < 0.001 vs*.* S100A9 group.

The mechanism by which S100A9 influences tight junction proteins in intestinal epithelial cells remains poorly understood. Research by Rinnerthaler et al. has demonstrated that low Ca^2+^ concentrations in the skin impairs barrier repair (Rinnerthaler et al., 2015). Therefore, we used the Fluo-4 a.m. calcium ion fluorescent probe to assess alterations in cellular calcium ion levels. As shown in Figures 6A,B, recombinant human S100A9 protein (1 μg/mL) treated significantly reduced intracellular Ca^2+^ concentrations. Pre-treatment of NCM460 cells with AT-1 indicated that AT-1 did not alter the decrease in intracellular Ca^2+^ levels induced by recombinant human S100A9 protein. Considering that Ca^2+^ concentration activates multiple signaling pathways that have known to regulate tight junctions, we measured the signaling transduction in MAPK, MLCK, and AMPK under recombinant human S100A9 protein and AT-1 treatment (Figures 6C–F). Notably, AT-1 did not influence the activation of ERK, JNK, P38; However, AT-1 enhances p-AMPK while inhibiting p-mTOR, with minimal effect on the MLCK signaling pathway.

**FIGURE 7:**
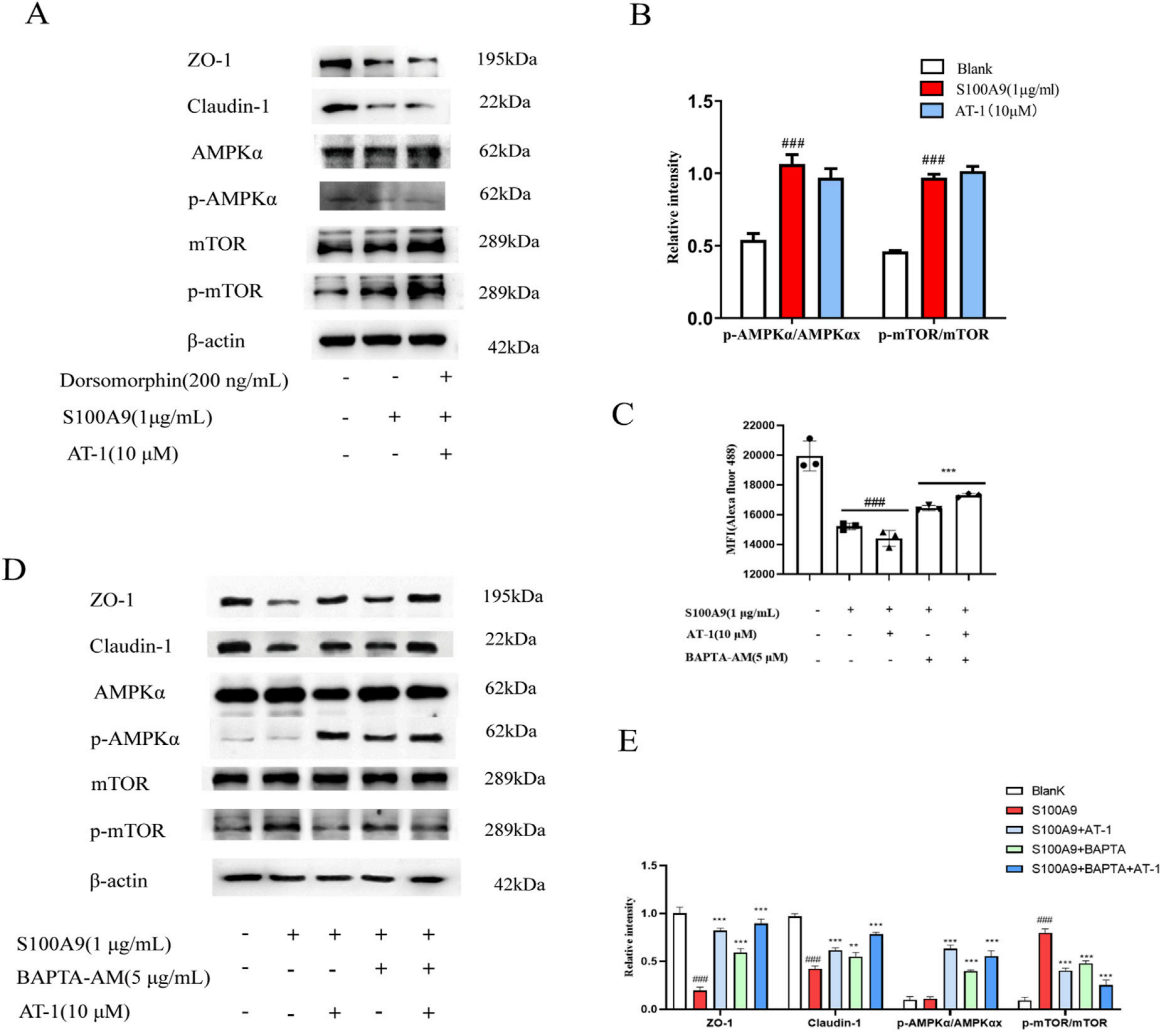
AT-1 regulated the expression of TJ proteins in colonic epithelial cells through AMPK/mTOR pathway. **(A,B)** NCM460 cells were pretreated with Dorsomorphin(200 ng/mL) and AT-1 (10 μM) for 1 h, followed with S100A9 (1 μg/mL) for 24 h, the level of ZO-1, claudin-1, AMPKα, p- AMPKα, mTOR, p-mTOR, was evaluated by Western blot. **(C)** NCM460 cells were pretreated with BAPTA-AM (5 μM) and AT-1 (10 μM) for 1 h, followed with S100A9 (1 μg/mL) for 24 h, then detected by flow cytometry assay. **(D,E)** NCM460 cells were pretreated with BAPTA-AM (5 μM) and AT-1 (10 μM) for 1 h, followed with S100A9 (1 μg/mL) for 24 h, the level of ZO-1, claudin-1, AMPKα, p- AMPKα, mTOR, p-mTOR, was evaluated by Western blot. ###P < 0.001 vs. Blank group; *p < 0.05 and ***p < 0.001 vs. S100A9 group.

To elucidate the role of the AMPK/mTOR pathway in epithelial barrier protection, we utilized the AMPK inhibitor dorsomorphin (200 nM) in combination with AT-1. As shown in Figures 7A,B, the attenuation of tight junction protein loss by AT-1 was reversed by dorsomorphin, evidenced by a decrease in p-AMPKα and an increase in p-mTOR. To further elucidate the role of calcium signaling in recombinant human S100A9 protein-mediated AMPK/mTOR pathway activation, we employed the calcium chelator BAPTA-AM to interrogate calcium-dependent mechanisms. Flow cytometry analysis demonstrated that BAPTA-AM significantly attenuated recombinant human S100A9 protein -induced depletion of intracellular Ca^2+^ concentrations (Figure 7C). Western blotting further revealed that BAPTA-AM restored the expression of tight junction proteins and normalized AMPK/mTOR signaling perturbations caused by recombinant human S100A9 protein exposure (Figures 7D,E). Strikingly, co-treatment with AT-1 and BAPTA-AM synergistically enhanced therapeutic outcomes.

These findings demonstrate that AT-1 modulates recombinant human S100A9 protein-mediated AMPK/mTOR signaling pathway in colonic epithelial cells, thereby influencing the expression of tight junction proteins.

## 4 Discussion

Ulcerative colitis (UC) is a commonly prevalent, chronic, non-specific inflammatory disease that primarily affects the mucosal and submucosal tissues of the colon and rectum (Gros and Kaplan, 2023). The pathogenesis of UC is complex, characterized by a protracted disease course that is challenging to cure and poses a potential increased risk of cancer (Shah and Itzkowitz, 2022). In recent years, the global incidence of UC has been growing rapidly, while current treatment strategies predominantly focus on anti-inflammatory therapies (Bruner et al., 2023; Le Berre et al., 2023). There is clinical recognition that inhibiting the overactivation of immune cells and strengthening intestinal barrier function may represent a novel therapeutic strategy (Odenwald and Turner, 2017). Our study has identified that AT-1, a key active component of *A. macrocephala* Koidz., exhibits a unique dual mechanism of action. On one hand, AT-1 effectively inhibits neutrophil activation, leading to a reduction in the release of S100A8/A9 proteins. On the other hand, AT-1 counteracts the loss of tight junction proteins induced by S100A9, thereby improving intestinal barrier dysfunction associated with DSS treatment. This discovery offers a new perspective and potential therapeutic avenue for the treatment of ulcerative colitis.

Due to its simplicity and high reproducibility, the DSS-induced ulcerative colitis model is widely employed in the development of colitis treatments, encompassing both acute and chronic models (Vlantis et al., 2016; Li et al., 2022). Oral administration *via* drinking water induces several features characteristic of flares seen in human ulcerative colitis (UC), including weight loss, bloody diarrhea, and ulcer formation. Stefan Wirtz has highlighted that DSS colitis is frequently utilized in studies investigating the role of innate immune mechanisms in the development of mucosal inflammation and the restoration of barrier integrity (Wirtz et al., 2017). Previous pharmacological study of AT-1 focused exclusively on *in vivo* results and analyses (Qu et al., 2022). However, the underlying mechanism was not extensively studied. To enhance our understanding of AT-1’s impact on the intestinal barrier, we adopted a model detailed in Figure 1A, which aligns more closely with the research of Stefan Wirtz and has been validated in our earlier studies (Lv et al., 2021a; Lv et al., 2021b), marking a significant departure from previous research. Our findings indicate that AT-1 accelerates the recovery of body weight in the mice, significantly reduces the Disease Activity Index (DAI) score, and histological assessments show that AT-1 promotes the healing of the intestinal mucosa. Further investigation reveals that AT-1 demonstrates enhanced therapeutic effects on the physical barrier, particularly in regulating tight junction proteins.

S100A9 can bind to S100A8 to form a heterodimer known as S100A8/A9, also referred to as calprotectin (calcium-binding protein, CP)(Parisien et al., 2022; Jakobsson et al., 2023). In the serum and colonic tissues of patients with UC, calprotectin is highly expressed, and its levels are closely associated with disease severity (Ricciuto and Griffiths, 2019; Khaki-Khatibi et al., 2020). Fecal calprotectin, which reflects the presence of calprotectin released from intestinal tissues into the feces during inflammation, has recognized as a progressive biomarker in the diagnosis, differential diagnosis, treatment planning, and prognostic assessment of UC (Pruenster et al., 2016). While numerous studies have highlighted the important role of S100A8/A9 in inflammatory responses, few have reported their direct interactions with intestinal epithelial cells. This study identifies for the first time that it is S100A9, rather than S100A8, that exerts a destructive effect on tight junction proteins, providing new insights into the pathogenesis of UC. Immunofluorescence staining experiments further demonstrated that AT-1 reduces the co-localization of CD11b^+^ neutrophils with S100A9, suggesting that AT-1 inhibits the release of S100A9 derived from neutrophils. Moreover, AT-1 mitigates the harmful effects of recombinant S100A9 protein on tight junction proteins in intestinal epithelial cells, confirming its potential value in protecting intestinal barrier function.

As a crucial second messenger within cells, Ca^2+^ plays an essential role in information transmission and the regulation of various cellular activities (Lee et al., 2012; Medina et al., 2015; Hernández-Oliveras and Zarain-Herzberg, 2024). Its relationship with cellular barriers has also been documented. Previous studies indicate that ion gradients, including calcium and magnesium, in the epidermis are vital for maintaining skin barrier homeostasis. Research by Propsond and colleagues utilized an *in vitro* blood-brain barrier (BBB) model to demonstrate intracellular Ca^2+^ not only as a downstream effector molecule in the C3a/C3aR signaling pathway, but also indispensable for maintaining vascular endothelial cadherin junctions and overall barrier integrity (Propson et al., 2021). Furthermore, David’s research highlighted that Ang1 effectively prevents VEGF-induced TRPC1-dependent Ca^2+^ influx by disrupting the interaction between IP3R and TRPC1, thus mitigating increases in endothelial permeability (Jho et al., 2005). In our study, we confirm that S100A9, as a calcium-binding protein, can reduce intracellular Ca^2+^ concentration in intestinal epithelial cells, suggesting a potential mechanism for its regulation of tight junction protein expression. Notably, changes in Ca^2+^ concentration are recognized by CaMK (Racioppi and Means, 2008; Reyes Gaido et al., 2023; Lisek et al., 2024), which triggers multiple signaling pathways. It has been demonstrated that these pathways can regulate MAPK (Parvathaneni et al., 2021), AMPK (Woods et al., 2005), and MLCK (Murthy, 2006) signaling, and alterations in these pathways have been reported to be associated with the dynamic regulation of tight junctions. Our findings indicate that Ca^2+^ changes induced by S100A9 activate these signaling pathways, while AT-1 regulates the expression of tight junction proteins by modulating the phosphorylation of AMPK and mTOR in response to altered Ca^2+^ concentrations.

Furthermore, previous studies have identified S100A9 as a mediator acting through TLR4 receptor activation (Ursino et al., 2022; Zhan et al., 2023), while AT-1 has been reported as a TLR4 inhibitor (Huang et al., 2014). However, our findings demonstrate that AT-1 treatment did not mitigate recombinant human S100A9 protein-induced alterations in intracellular calcium levels, suggesting the existence of a novel S100A9 signaling mechanism independent of TLR4. Subsequent investigation into calcium dynamics revealed that AT-1 modulates the AMPK/mTOR pathway, though its target specificity remains unclear. To further explore calcium-mediated signaling in tight junction regulation, we employed the calcium chelator BAPTA-AM. Strikingly, BAPTA-AM significantly attenuated recombinant human S100A9 protein-triggered decline in intracellular calcium concentrations and enhanced the expression of tight junction proteins in NCM460 cells. Notably, co-administration of AT-1 with BAPTA-AM synergistically amplified tight junction protein expression, highlighting a potential therapeutic strategy for restoring epithelial barrier integrity.

## 5 Conclusion

In summary, this study demonstrates that AT-1 protects intestinal barrier function and alleviates symptoms of DSS-induced colitis by inhibiting neutrophil activation and the transcription of S100A8/A9. Additionally, *in vitro* models further confirm that AT-1 mitigates the alterations in the AMPK/mTOR signaling pathway in intestinal epithelial cells induced by S100A9 protein, thereby reducing the loss of tight junctions (TJs). These findings offer new insights into the therapeutic potential of AT-1 for the treatment of ulcerative colitis and highlight its role in protecting intestinal barriers, laying a solid foundation for future research.

## Data Availability

The original contributions presented in the study are included in the article/supplementary material, further inquiries can be directed to the corresponding authors.
