# Supplementary figures and images for: Atractylenolide-I restore intestinal barrier function by targeting the S100A9/AMPK/mTOR signaling pathway
Source: Front Pharmacol. 2025 Mar 24;16:1530109. doi: 10.3389/fphar.2025.1530109 (PMC11973269; doi:10.3389/fphar.2025.1530109)

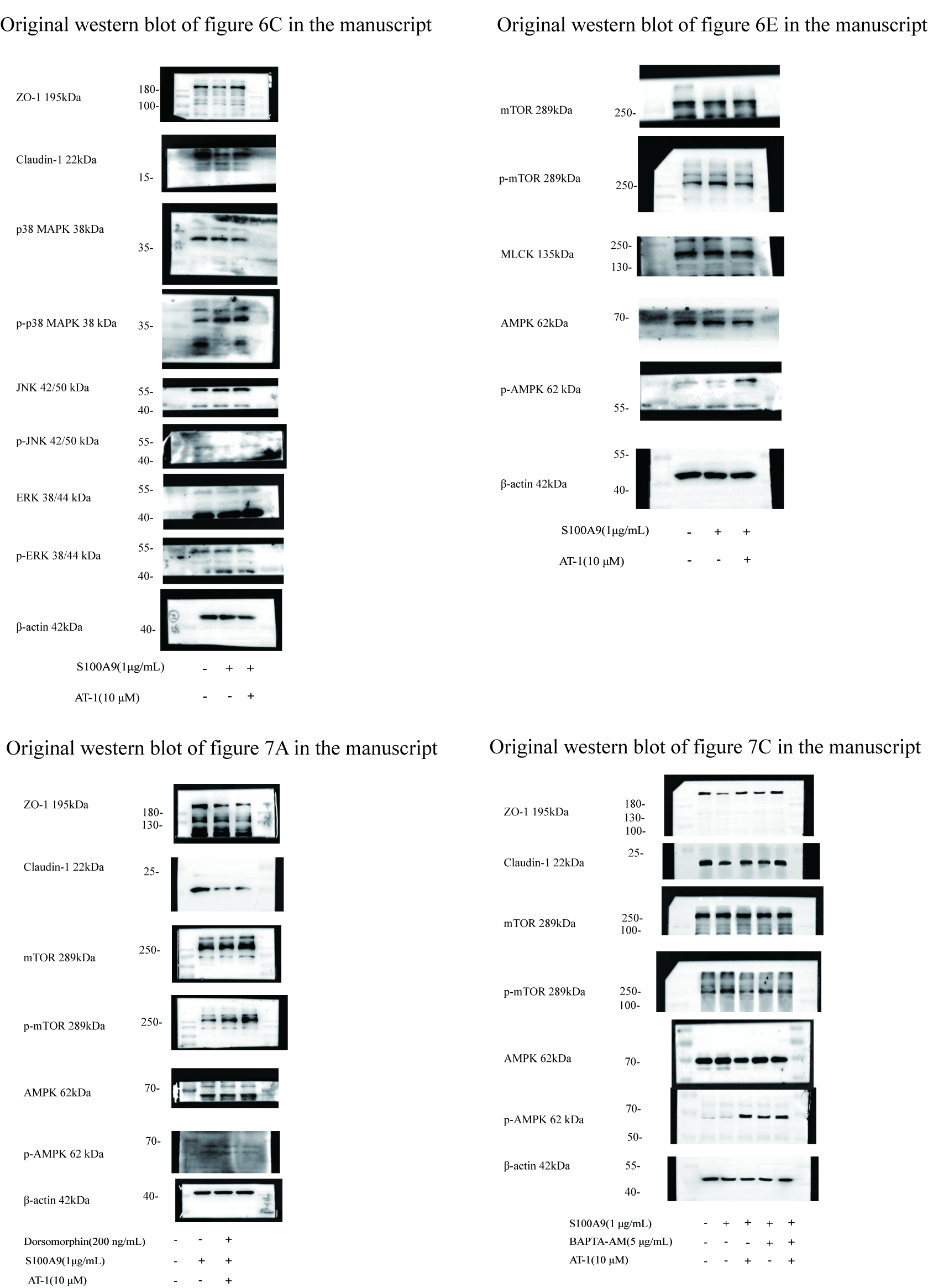

Supplement: Supplementary file 1 [file Image2.TIF]

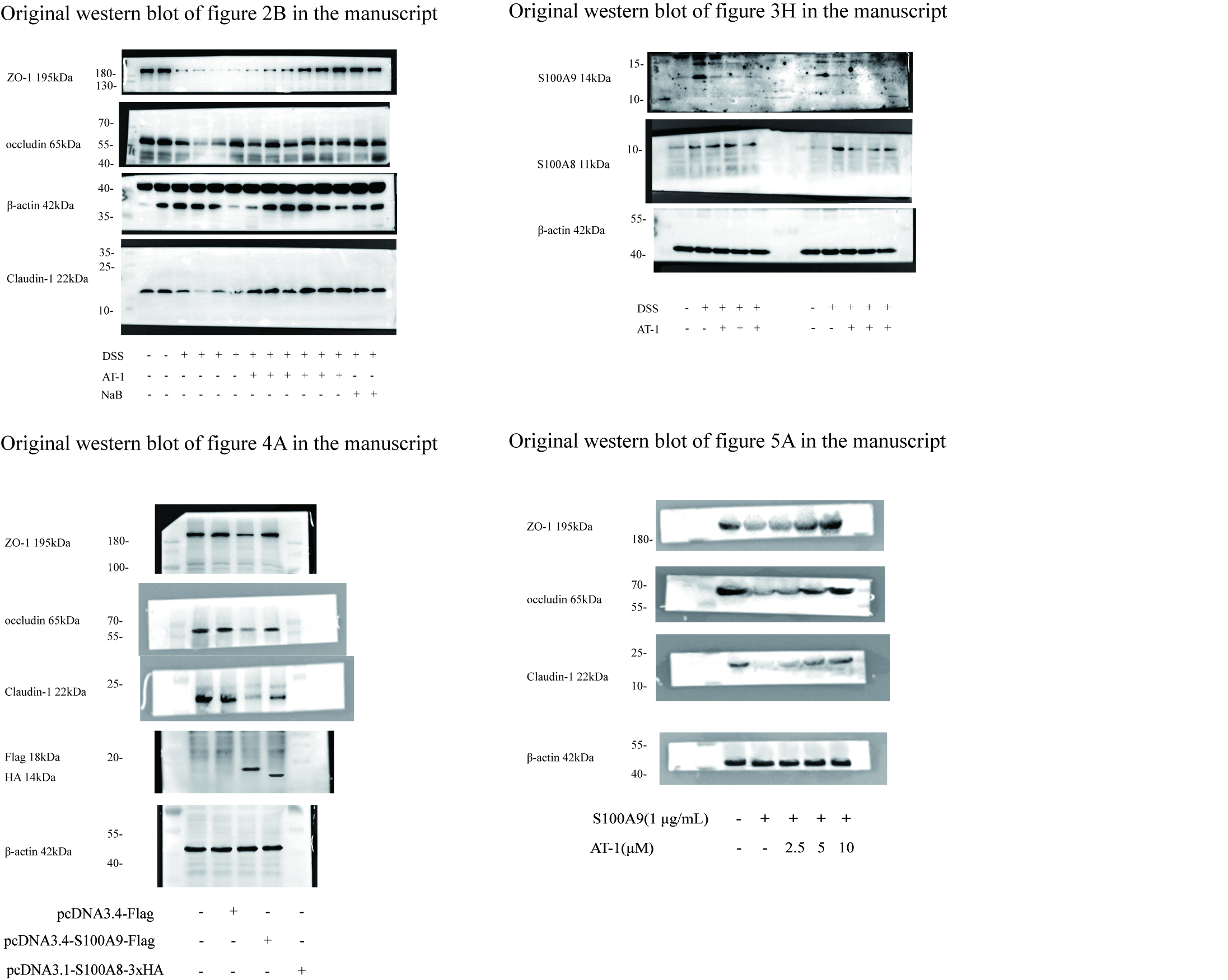

Supplement: Supplementary file 2 [file Image1.TIF]
